# Study of the Antidiabetic Activity of *Punica granatum L.* Fruits Aqueous Extract on the Alloxan-Diabetic Wistar Rats

**Published:** 2019

**Authors:** Ehsan Gharib, Shideh Montasser Kouhsari

**Affiliations:** *Department of Cellular and Molecular Biology, School of Biology, University College of Science, University of Tehran, Tehran, Iran.*

**Keywords:** Alloxan monohydrate, Diabetes mellitus, Insulin expression and secretion, Insulin signaling mediators, Glucose uptake and storage, Pomegranate fruits aqueous extract

## Abstract

Pancreatic β-cells dysfunction and impairment of insulin action usually leads to hyperglycemia. *Punica granatum L.* is a well-known traditional herbal remedy in Iran due to its positive effects on ameliorating blood glucose homeostasis. In this study, Alloxan-diabetic male Wistar rats were administrated with pomegranate fruits aqueous extract (PE) in different doses of 100, 200, and 350 mg/kg bw (PE+Da, PE+Db and PE+Dc, respectively), and the effects of PE polyphenols content on glucose metabolism in treated groups were examined using oral glucose tolerance test (OGTT), short-term and long-term PE consumption periods models followed by evaluation of plasma insulin, free fatty acids, and triglycerides levels and tissues contents of glycogen and triglycerides; compared with diabetic control (DC) and healthy control (NC) groups. By using Real-time PCR, the possibility of modulations of the Insulin receptor substrate 1 (IRS-1), Protein kinase B (Akt), Glucose transporter 2 and 4 (Glut-2, 4) mRNAs expression levels in PE treated rats were investigated. The obtained data showed noticeable reduction in fasting blood glucose (FBG) by 28.1% and 67.9% in short-term and long-term treatment models, respectively, in PE + Dc group. Also, there existed marked increase in the mRNAs expression levels of IRS-1, Akt, Glut-2, and Glut-4, which results in improvement of glucose uptake and promotes its storage. Taking together, it is suggested that PE administration contributes to the modulation of both hyperglycemia and hyperlipidemia in Alloxan-diabetic Wistar rats.

## Introduction

Diabetes mellitus (DM) is one of the most common metabolic diseases in the world that results from defects in endogenous insulin secretion/action and thereby contributes to the impairment of glucose disposal (hyperglycemia) (1). Chronic hyperglycemia triggers severe diabetic complications such as dysfunction of insulin-stimulated glycogen synthesis and increased glucose output (2).

Pomegranate (*Punica granatum L.*) appears to be native to some parts of Asia (Iran, Malesia, and India), America (USA, Peru), Africa (Equatorial region), and Europe (Turkey). The fruits are consumed mostly fresh or in the form of derived productions like juice, paste, jam, and wine (3). Fresh pomegranate juice has a high polyphenolic content such as punicalagin (the most abundant component), anthocyanin, phenolic acids, non-phenolic acids, tannins, and glutenins (4). 

Antidiabetic effects of pomegranate are especially because of its phenolic compounds and their potential abilities to act as highly effective agents in limiting the risk factors for diabetes. Studies have examined the action of pomegranate extracts in preventing obesity (5) and hyperlipidemia (6). However, the underlying hypoglycemic mechanism of the polyphenolic contents of pomegranate has not been fully elucidated yet. The present study investigated the molecular and biochemical effects of pomegranate fruits aqueous extract on Alloxan-induced diabetic rats.

## Experimental

*Chemicals and reagents*

Alloxan monohydrate was supplied by Sigma (St. Louis, MO, USA); the RNA extraction kit was taken from SinaClon Co. (Tehran, Iran); the Prime Script RT Reagent Kit was obtained from TaKaRa Biotechnology (Tokyo, Japan); the Super SYBR Green qPCR Master Mix 2x was bought from Yekta Tajhiz Co. (Tehran, Iran); Rat Insulin ELISA kit was purchased from Mercodia (Sweden); the Triglyceride Quantification Assay kit was taken from Abcam (USA); the commercial Free Fatty Acid Assay kit was obtained from Enzychrom Bio-Assays Systems (USA) and the commercial glycogen assay kit was supplied from Biovision (Mountain View, CA). All other chemicals and solvents were of the highest commercial grade and purchased from either Merck (KGaA, Germany) or Sigma (St. Louis, MO, USA).

*Animals*

Male Wistar rats (*Rattus norvegicus*) weighing 180–220 g were purchased from the School of Pharmacy, Tehran University of Medical Sciences (Tehran, Iran). The animals were housed 4 per standard rat cage in a room with a 12:12 h light/dark cycle and controlled temperature (24 ± 1 °C) and were allowed to adapt themselves to the new location for one week. They were provided with water and a standard diet (27% protein, 32% fat, and 41% carbohydrate) ad libitum.

All procedures involving animals and their care (including Feeding, Extract administrating, Blood sampling, Gastric intubation, Anesthesia and Euthanasia) were under the close supervision of qualified and experienced personnel and were approved by the Institutional Animal Care and Use Committee of University of Tehran.

*Preparation of the pomegranate fruits aqueous extract*

Pomegranate fruits were washed and manually peeled without separating the seeds. The extract was obtained using a blender, filtered through Whatman No. 1 filter paper to remove any water insoluble materials, and steamed (5 min) for enzyme inactivation. It was then stored at −18 °C. Extract underwent a drying process in a cabinet dryer at 37 °C for 72 h and was suspended in distilled water at doses of 100, 200, and 350 mg/kg body weight (bw).

*Preparation of the Alloxan-induced diabetic Wistar rats*

Diabetes was induced in the Wistar rats after overnight fasting by injecting Alloxan monohydrate (120 mg/kg) dissolved in citrate buffer (pH 4.5) subcutaneously. Hyperglycemia was confirmed by the elevated glucose levels in the surviving rats’ blood, determined at 72 h after injection using blood samples obtained from the tail vein using a glucometer device (On Call Now, San Diego, USA). The rats with a blood glucose level of ≥ 300 mg/dL were considered to be diabetic.

*Experimental design*

The rats were randomly divided into eight groups, each consisting of 12 rats, and placed in cages (two rats per cage). They were treated orally with PE for a period of 21 days as follows:

Group I (NC): Normal rats treated with vehicle alone;

Group II (PE +Na): Normal rats treated with PE at a dose of 100 mg/kg bw.

Group III (PE +Nb): Normal rats treated with PE at a dose of 200 mg/kg bw.

Group IV (PE +Nc): Normal rats treated with PE at a dose of 300 mg/kg bw.

Group V (DC): Diabetic rats treated with vehicle alone;

Group VI (PE+Da): Diabetic rats treated with PE at a dose of 100 mg/kg bw.

Group VII (PE+Db): Diabetic rats treated with PE at a dose of 200 mg/kg bw.

Group VIII (PE+Dc): Diabetic rats treated with PE at a dose of 350 mg/kg bw.

All rats were provided with water and a standard diet ad libitum. 

The food and water were removed from cages 12 h before testing. Using an intragastric tube, 1 ml of predetermined doses was administered orally to rats and postprandial blood glucose (PBG) was estimated in a short-term model (1, 3, 5, 8 and 24 h).

OGTT was performed on all animals one week before they were euthanized. All groups received a carbohydrate solution (maltose and sucrose each 1 g/kg bw) through gastric intubations, and PBG was monitored at the time: 0 (before-glucose load) and at 30, 60, 90, and 120 min after carbohydrate solution administration. At the end of the experimental period, the animals were fasted overnight and the blood samples were collected from the rats via intra-cardiac puncture (under anesthesia) on day 21. The blood samples were collected in both ordinary and lithium heparin tubes. For serum separation, the blood samples were incubated at room temperature to allow the blood to clot and then centrifuged at 1000 g for 15 min to separate the plasma from erythrocytes. The serums were stored in aliquots at −70 °C for biochemical analysis. The tissues were collected, weighed, and frozen in liquid nitrogen and stored 

at −180 °C.

*Biochemical analysis*

Fasting glycemia, lipidemia and insulinemia were monitored in the morning of the blood sample collection. Serum glucose was determined using a glucometer device. Plasma triglyceride (TG) and free fatty acid (FFA) concentrations were quantified using the Triglyceride Quantification Assay kit (Abcam) and Free Fatty Acid Assay kit (Enzychrom Bio-Assays Systems) according to the manufacturers’ guidelines. Plasma insulin was detected using a rat insulin ELISA kit (Mercodia).To determine the TG level in the liver, heart and skeletal muscles, the samples were resuspended and homogenized in 1 mL of 5%NP-40/ddH2O solution, boiled and later centrifuged at 12,000 g for 2 min; TG concentrations were analyzed with the same kit as used in the plasma analysis.

To determine the glycogen content in the same tissues, the samples were dissolved in 30% KOH, boiled and later centrifuged at 12,000 g for 5 min; glycogen was measured in the supernatants using the commercial glycogen assay kit.

*RNA extraction and quantitative reverse transcription-polymerase chain reaction*

Total RNA was extracted from homogenized suspension of target tissues using a Polytron PT1600E bench-top homogenizer (Kinematica AG, Switzerland) and RNX-Plus reagent kit according to the manufacturer’s protocol. 

The RNA concentration was measured by a Nanodrop ND-1000 spectrophotometer (Nanodrop Technologies), and the quality was assayed by an A260/A280 ratio of 1.8–2.0. 1 µg of total RNA were reverse-transcribed with the Prime Script RT reagent kit. Quantitative real-time reverse transcription-PCR was carried out on an ABI Step One RT-PCR thermal cycler (ABI Step one, NY, USA) using a YTA SYBR green qPCR master Mix 2X kit according to the manufacturer’s instructions. Primer sequences were designed for all genes using PerlPrimer and Primer-BLAST (NCBI) was used to check their specificity. Ribosomal 18S RNA was utilized to calculate the relative abundance of mRNA transcripts. To assess RNA integrity, Ribosomal 28S RNA levels were also measured. Each measurement was performed in triplicate. Primer sequences of Real-time PCR are listed in Table 1.

*Data analysis*

All data are represented as the mean ± S.E.M (Standard Error Mean). Comparisons between groups were made by One-way analysis of variance (ANOVA) followed by an appropriate Post-hoc test to analyze the difference. Statistical significance was achieved when *P* < 0.05.

## Results and Discussion

In diabetic condition, insulin secretion and action are considerably reduced and results in hyperglycemia. Insulin hormone increases Glut-2 and Glut-4 mRNAs expression and proteins translocation to plasma membranes in tissues (7). Subsequently, insulin regulates mediators which are involved in TG and glycogen synthesis, gluconeogenesis and glycolysis (2, 8). The defect in insulin stimulated pathways contributes to constant hyperglycemia and results in impairment of glucose disposal and enhances glucose output (9). 

Alloxan is an oxygenated pyrimidine and the toxic analog of glucose, which selectively uptakes via Glut-2 in pancreatic β-cells and causes deficiency of insulin secretion and glucose disposal and also enhances hyperglycemia (10). In this study, as demonstrated in Table 2, an elevated blood glucose level is observed in diabetic rats (20.92 ± 2.7 mmol/L) after injecting 120 mg/kg bw Alloxan monohydrate.

Our results indicate that PE administration normalizes glycemia, as assessed by OGTT experiment.

The pomegranate polyphenols extract is proven to reduce the glucose levels on diabetic groups (11, 12). The PBG of each animal was determined at 0 min, just before carbohydrate solution loading, and at 30-, 60-, 90- and 120-min time points. As shown in Table 3, PBG concentrations increased 30 min after carbohydrate solution administration in all groups and subsequently decreased. The increment in PBG level at 30 min was significantly suppressed in the PE+D groups compared with the DC group (*P* < 0.001). There were also differences in PBG values between the treated groups and the DC group at 90 and 120 min after carbohydrate solution administration (*P* < 0.001). The improvement in OGTT might have been due to the suppression of glucose intestinal absorption by anthocyanin (13) and quercetin (14), which could contribute to the post-prandial glycemic control and body weight gain.

PE administration had a positive effect on short-term and long-term blood glucose levels in Alloxan diabetic rats, which is associated with enhanced β-cell function and decreasing FBG as previously reported *in-vitro* (15) and *in-vivo* (16). Table 2 illustrates the antihyperglycemic effects of PE on PBG levels in diabetic and normal rats, after a single dose administration. Serum glucose was measured before (−5 min) and at 1, 3, 5, 8 and 24 h after taking the extract at the doses of 100, 200 and 350 mg/kg bw. In PE treated groups, a reduction in PBG was observed after 1 h till 8 h compared to DC group (*P* < 0.05). Also, glucose concentrations were measured in target groups after 7, 14, and 21 days (Table 4). In DC rats, the FBG value was increased significantly; whereas the proportion of FBG in PE treated individuals were reduced 64.78% and 67.95% compared to initial day and DC controls, respectively (*P *< 0.001).

**Figure 1:**
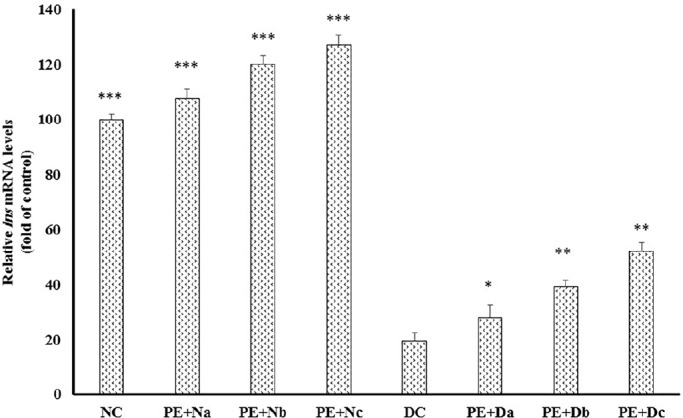
Real-time polymerase chain reaction (PCR) of the mRNA expression levels of insulin**, **in the non-diabetic control group (NC, n = 12), non-diabetic group (a, b and c) treated with PE (PE + N, n = 12), diabetic control group (DC, n = 12), and diabetic group (a, b and c) treated with PE (PE+D, n = 12). Value ratios are expressed as a percentage relative to DC rats. 18s RNA was used as an internal control (n = 3). The mean of six independent experiments is shown. *Significantly different from DC group

DC rats showed significant reduction in *ins* expression compared to normal controls (Figure 1); in contrast, daily PE intake amplified insulin mRNA levels about 3 to 3.5 fold in PE + Dc 

(*P* < 0.001). Also, there was a marked reduction in the level of serum insulin in the diabetic group in comparison to healthy models (Table 5), whereas PE administration elevated insulin production/secretion in PE + Db and PE + Dc about 46.5% (*P* < 0.05) and 74.41%, respectively (*P* < 0.01). In line with these results, several researches have been dedicated to the promoting effects of polyphenolic constituents, which are also present in pomegranate extract, on the plasma insulin levels. Gallic acid, as an important constituent of pomegranate, is shown to increase plasma insulin in Streptozotocin-induced diabetic rats (15, 16). Oral administration of ellagic acid, another pomgeranate polyphenlic compound, elevates insulin production and causes blood glucose level reduction in diabetic mice (17). In addition, studies investigate the effects of quercetin on regeneration of the pancreatic islets and insulin secretion in Streptozotocin-induced diabetic rats (18-20). Together with these previous findings, our study shows that PE administration promotes insulin mRNA expression (Figure 1) and insulin secretion (Table 5) from pancreatic β-cells and improves the glycemic control, in Alloxan-induced diabetic rats.

**Table 1:** Primer sequences used for Real-time PCR

**Primer**	**Sequence (5’–>3’)**
*irs-1*	GAT ACC GAT GGC TTC TCA GAC G
	TCG TTC TCA TAA TAC TCC AGG CG
*akt*	GAA GCT GAG CCC ACC TTT CA
	CAT CTT GAT CAG GCG GTG TG
*glut-2*	GTC CAG AAA GCC CCA GAT ACC
	TGC CCC TTA GTC TTT TCA AGC T
*glut-4*	TTC TGT TGC CCT TCT GTC CTG AGA G
	GAG CAC CGA GAC CAA CGT GAA GAC
18S	GGA CAC GGA CAG GAT TGA CA
	ACC CAC GGA ATC GAG AAA GA
28S	GGT AAA CGG CGG GAG TAA CTA TG
	TAG GTA GGG ACA GTG GGA ATC TCG

**Table 2:** Glycemic control in Alloxan-diabetic rats during 24 h treatment with PE

**Groups**	**Dose (mg/kgbw)**	**Fasting blood glucose (mmol/L)** **Days after a single dose daily treatment**					
		**0**	**1**	**3**	**5**	**8**	**24**
NC	-	4.92±3.1^f^	5.31±2.5^f^	6.17±2.8^a^,^f^	5.74±2.6^a^,^f^	4.77±2.7^f^	5.53±2.3^a^,^f^
PE+N^a^	100	4.91±1.6^f^	5.13±3.5^f^	5.25±1.1^f^	5.17±1.4^f^	4.91±3.1^f^	5.27±1.5^f^
PE+N^b^	200	4.92±2.3^f^	5±2.6^f^	5.12±1.8^f^	5.04± 2.4^f^	4.86±2.7^f^	5.1±2.7^f^
PE+NC	350	4.92±2.1^f^	4.73±1.3^f^	4.94±1.5^f^	4.82±1.1^f^	4.4±2.1^f^	4.96±2.2^f^
DC	-	20.92±2.7	21.37±3.3	24.9±3.7^c^	24.3±2.9^c^	25.2±2.6^c^	25.9±3.1^c^
PE+D^a^	100	20.81±3.9	20.32±1.3^e^	20.19±1.9^f^	20.01± 2^f^	19.33±1.8^a^,^f^	20.23±1.9^f^
PE+D^b^	200	20.79±2.5	19.33±3.6^a^,^d^	19.08±3.7^a^,^f^	18.84 ± 4^b^,^f^	18.14±3.8^b^,^f^	19.47±3.5^a^,^f^
PE+D^c^	350	20.87±2.8	18.11±2.5^b^,^f^	17.61±1.7^c^,^f^	17.25±1.1^c^,^f^	16.43±1.6^c^,^f^	18.62±2.1^b^,^f^

NC, normal control; PE+NC (a, b and c); DC, diabetic control; PE+D (a, b and c), diabetic rats treated with 100, 200 and 350 mg/kg bw, respectively of pomegranate fruits aqueous extract. Each value is the mean±S.D. of six separate experiments.

a *P *< 0.05 vs. time 0. *P *< 0.01 vs. time 0. *P *< 0.001 vs. time 0. *P *< 0.05 vs. DC.

e *P *< 0.01 vs. DC.

f *P *< 0.001 vs. DC.

**Table 3:** Effect of PE on oral glucose tolerance test

**Groups**	**Dose (mg/kg bw)**	**Fasting blood glucose (mmol/L)** **Days after a single dose daily treatment**				
		**0**	**30**	**60**	**90**	**120**
NC	-	4.92±3.4^f^	7.58 ± 2.9^b^,^f^	7.08 ± 3.3^b^,^f^	5.88 ± 2.1^f^	4.96 ± 3.2^f^
PE+N^a^	100	4.6±1.5^f^	7.26±1.1^b^,^f^	7.12±1.3^b^,^f^	5.13±2^f^	4.7±2.4^f^
PE+N^b^	200	4.2±1.7^f^	6.93±1.6^a^,^f^	6.41±1.2^a^,^f^	4.7±1.5^f^	4.2±1.1^f^
PE+NC	350	4.3±1.3^f^	6.2±1.2^a^,^f^	5 ±1.7^f^	4.3±1.2^f^	4.3±1.4^f^
DC	-	23.19±2.1	35.45±1.1^c^	33.41±2.6^c^	32.07±4.3^c^	31.15±2.5^c^
PE+D^a^	100	19.77±2.6^f^	33.47 ± 1.9^c^,^f^	30.43 ± 2.2^c^,^f^	27.92 ± 2.3^c^,^f^	24.91 ± 2.9^c^,^f^
PE+D^b^	200	15.48±1.8^f^	28.71±3.5^c^,^f^	25.16±3.8^c^,^f^	22.38±3.7^c^,^f^	17.45±3.2^b^,^f^
PE+D^c^	350	11.37±2.4^f^	22.31 ± 2.6^c^,^f^	18.44 ± 2.1^c^,^f^	14.39 ± 2.3^b^,^f^	12.72± 2.7^a^,^f^

NC, normal control; PE+NC (a, b and c); DC, diabetic control; PE+D (a, b and c), diabetic rats treated with 100, 200 and 350 mg/kg bw, respectively of pomegranate fruits aqueous extract. Each value is the mean±S.D. of six separate experiments.

a *P *< 0.05 vs. time 0. *P *< 0.01 vs. time 0. *P *< 0.001 vs. time 0. *P *< 0.05 vs. DC.

e *P *< 0.01 vs. DC.

f *P *< 0.001 vs. DC.

**Table 4.** Glycemic control in Alloxan-diabetic rats during 3 weeks treatment with PE. FBG was measured at the end of first, second and third weeks (long-term period).

**Groups**	**Dose (mg/kg bw)**	**Fasting blood glucose (mmol/L)** **Days after a single dose daily treatment**			
		**0**	**7**	**14**	**21**
NC	-	4.92 ± 3.1^d^	4.91 ± 2.9^d^	4.92 ± 3.4^d^	4.94 ± 3.2^d^
PE+N^a^	100	4.91±1.6^f^	4.7 ± 1.3^f^	4.6 ± 1.5^f^	4.5 ± 2.1^f^
PE+N^b^	200	4.92±2.3^f^	4.5 ± 2.2^f^	4.2 ± 1.7^f^	4.1 ± 1.9^f^
PE+NC	350	4.92±2.1^f^	4.3 ± 1.2^f^	4.3 ± 1.3^f^	4.2 ± 1.5^f^
DC	-	20.92 ± 2.7	21.33 ± 2.3	23.19 ± 2.1^b^	22.93 ± 3^b^
PE+D^a^	100	20.81 ± 3.9	21.29 ± 1.7	19.77 ± 2.6^a^,^f^	17.7 ± 1.2^b^,^f^
PE+D^b^	200	20.79 ± 2.5	18.92 ± 2.2^a^,^e^	15.48 ± 1.8^c^,^f^	12.66 ± 2.7^c^,^f^
PE+D^c^	350	20.87 ± 2.8	16.93 ± 2^c^,^f^	11.37 ± 2.4^c^,^f^	7.35 ± 2.3^c^,^f^

NC, normal control; PE+NC (a, b and c); DC, diabetic control; PE+D (a, b and c), diabetic rats treated with 100, 200 and 350 mg/kg bw, respectively of pomegranate fruits aqueous extract. Each value is the mean±S.D. of six separate experiments.

a *P *< 0.05 vs. time 0. *P *< 0.01 vs. time 0. *P *< 0.001 vs. time 0. *P *< 0.05 vs. DC.

e *P *< 0.01 vs. DC.

f *P *< 0.001 vs. DC.

**Table 5:** Clinical characteristics of studied groups

**Groups**	**NC**	**PE + N** a	**PE + N** b	**PE + N** c	**DC**	**PE + D** a	**PE + D** b	**PE + D** c
Variables Insulin (ng/mL)	11.42 ± 1.8	11.90 ± 1.2	12.43 ± 1.1^a^	13.73 ± 1.4^b^	4.3 ± 1.1^c^	5.1 ± 2.6^c^	6.3 ± 1.42^c^	7.5 ± 1.1^b^
Plasma FFA (mg/mL)	74.93 ± 2.1	73.58 ± 1.8	71.92 ± 2.3	69.44 ± 1.1	167.37 ± 2.9^c^	154.63 ± 2.3^c^	129.08 ± 1.8^b^	97.63 ± 2.4^a^
Plasma TG (mg/mL)	82.31 ± 1.9	81.17 ± 1.2	79.77 ± 1.4	75.28 ± 1.5	193.19 ± 2.2^c^	180.75 ± 1.2^c^	164.16 ± 1.1^c^	113.82 ± 1.2^a^
Initial body weight (g)	198.09 ± 2.1	198.38 ± 2.1	198.66 ± 2.3	198.49 ± 1.9	198.37 ± 2.2	198.13 ± 1.8	198.72 ± 2.3	198.13 ± 1.9
Final body weight (g)	202.29 ± 3.3	205.16 ± 2.8	204.53 ± 2.1	204.83 ± 1.3^a^	172.82 ± 3.2^c^	181.93 ± 2.3^c^	189.1 ± 2.1^b^	195.36 ± 2.4^a^
Skeletal muscle glycogen (mg/g)	20.11 ± 4.2	26.68 ± 3.5^a^	33.97 ± 2.9^b^	42.73 ± 2.8^c^	8.28 ± 2.17^c^	10.67 ± 3.73^c^	13.82 ± 3.19^c^	17.29 ± 3.5^b^
hepatic glycogen (mg/g)	89.19 ± 2.7	92.05 ± 1.1	99.7 ± 2.1^a^	117.82 ± 3.7^b^	19.74 ± 2.82^c^	24.13 ± 5.35^c^	44.81 ± 4.7^c^	60.77 ± 3.19^b^
Cardiac glycogen (mg/g)	14.82 ± 2.2	15.19 ± 3.5	16.23 ± 1.9^a^	22.93 ± 4.1^c^	6.11 ± 2.7^c^	7.38 ± 1.2^c^	8.19 ± 2.2^c^	11.87 ± 1.3^b^
Skeletal muscle TG (mg/g)	12.9 ± 1.9	13.2 ± 1.8	14.4 ± 2.4^a^	16.01 ± 1.3^c^	4.82 ± 2.7^c^	5.97 ± 1.9^c^	8.72 ± 3.7^c^	10.82 ± 2.6^b^
hepatic TG (mg/g)	17.5 ± 2.5	19.63 ± 2.8^a^	26.06 ± 3.3^c^	39.12 ± 2.1^c^	7.19 ± 1.2^c^	8.33 ± 3.7^c^	11.84 ± 3.1^b^	14.42 ± 2.9^a^
Cardiac TG (mg/g)	25.7 ± 3.1	26.1 ± 3.7	28.51 ± 3.1^a^	30.19 ± 1.4^c^	11.29 ± 2.4^c^	13.11 ± 1.9^c^	15.22 ± 1.2^c^	19.31 ± 2.5^b^

NC, normal control; PE+NC (a, b and c); DC, diabetic control; PE+D (a, b and c), diabetic rats treated with 100, 200 and 350 mg/kg bw, respectively of pomegranate fruits aqueous extract. Each value is the mean ± S.D. of six separate experiments.

a *P* < 0.05 vs. NC.

b *P* < 0.01 vs. NC.

c *P* < 0.001 vs. NC.

**Figure. 2:**
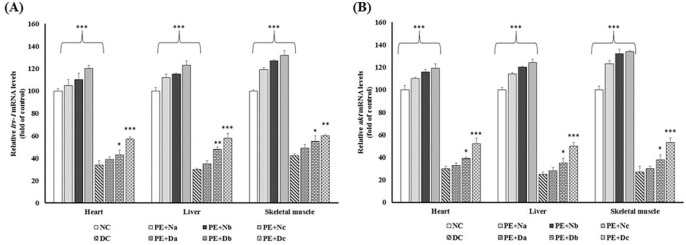
Real-time PCR of the mRNA expression levels of (A) IRS-1 and (B) Akt, in the non-diabetic control group (NC, n = 12), non- diabetic group (a, b and c) treated with PE (PE + N, n = 12), diabetic control group (DC, n = 12), and diabetic group (a, b and c) treated with PE (PE+D, n = 12). Value ratios are expressed as a percentage relative to DC rats. 18s RNA was used as an internal control (n = 3). The mean of six independent experiments is shown. *Significantly different from DC group

**Figure 3:**
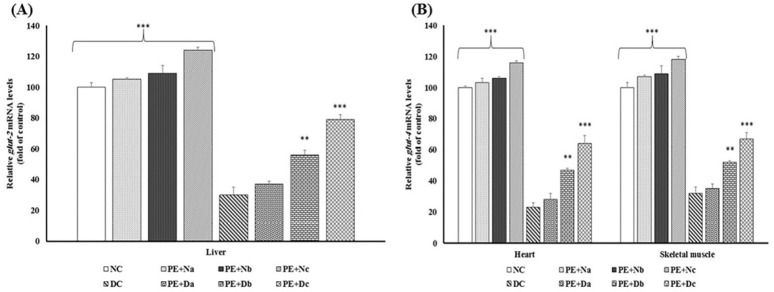
Real-time PCR of the mRNA expression levels of (A) Glut-2 and (B) Glut-4, in the non-diabetic control group (NC, n = 12), non-diabetic group (a, b and c) treated with PE (PE+N, n = 12), diabetic control group (DC, n = 12), and diabetic group (a, b and c) treated with PE (PE+D, n = 12). Value ratios are expressed as a percentage relative to DC rats. 18s RNA was used as an internal control (n = 3). The mean of six independent experiments is shown. *Significantly different from DC group

The reduction in the IRS-1 mRNA expression level is one of the major pathological mechanisms of diabetes. IRS-1 has a vital role in the insulin-stimulated glucose metabolism pathway and a defect in its expression leads to the disturbance of glucose metabolism through changes in downstream enzymes and kinases activities (17). The current study shows that there were noticeable differences in the level of IRS-1 mRNA between Alloxan diabetic and healthy control rats (Figure 2A), whereas in PE treated rats IRS-1 expression was promoted (*P* < 0.001). Due to the enhancement of *irs-1* expression in PE-treated rats compared with diabetic controls, it could be suggested that the hypoglycemic effects of PE might benefit from recovering the transcription ratio of IRS-1 in diabetic rat livers, as was shown previously in high-fructose diet rats (21) and insulin-resistant rats (22) which were administrated with flavonoid-rich extract of green tea .

Phosphoinositide 3-kinase (PI3k)/Akt pathway, which is located in the downstream of IRS-1, has a central role in insulin signaling. The activated PI3K upregulates Akt phosphorylation on residues Thr 308 and Ser 473 (23, 24). Akt regulates several metabolic processes. As shown in (Figure 2B), there was a reduction in Akt mRNA content in DC rats in comparison with NC group. Many studies have been carried out on the anti-diabetic effects of the flavonoids on restoring the impairment of PI3K/Akt pathway and elevating glucose uptake and storage, in both *in-vivo* (25-27) and *in-vitro* (26, 28, 29). Consistent with these reports, our study emphasizes this fact that prolonged PE treatment is contributed with the accelerating level of Akt mRNA expressions (Figure 2B).

Decrement of Akt expression/activity results in low rate of *glut-2* and *glut- 4* mRNAs expression and these proteins translocations from intracellular storage vesicles to the plasma membrane and thereby poor glucose uptake (30). Glut-2 and Glut-4 are key proteins involved in glucose uptake and storage in liver, heart and skeletal muscles (31). To study the glucose homeostasis, the mRNA expression of Glut-2 and Glut-4 were evaluated in all groups (Figure 3). 

The data depicted enhanced amount of glucose transport mediators’ mRNA in PE treated animals, whereas in the DC group, there were marked reductions in transcription levels of Glut-2 and Glut-4 (*P* < 0.001).

Suppression of Akt activity blocks glycogen synthase kinase 3 (GSK-3)/ Glycogen synthase (GS) pathway was followed by increments in glycogen synthesis (32). Also, Akt regulates phosphoenolpyruvate carboxykinase (PEPCK) and glucokinase (GK) suppression which are responsible for hepatic gluconeogenesis and glycolysis (2, 33). Moreover, low level of Akt, attenuates lipids synthesis because of down-regulation of transcription factors expressions and activities such as Sterol regulatory element-binding protein 1 (SREBP-1c) (34) and enhancement of lipolysis by inhibition of phosphodiesterase (PDE) enzyme and activation of hormone sensitive-lipase (HSL). As demonstrated in Table 5, plasma FFA and TG levels were raised in the diabetic controls in comparison with healthy models (Table 5). In contrast, the prolonged PE treatment showed promising reduction in FFA and TG concentration compared to diabetic controls, which in PE + Dc rats the differences were significant (*P *< 0.001). Also, DC group had the lowest levels of TG and glycogen storages (*P* < 0.001), whereas PE administration increased TG level and glycogen storage in diabetic rats, bringing it close to the levels of the NC group. Moreover, PE intake causes an enhancement in the body weight of Wistar rats without affecting the food intake (Table 5), due to increase of insulin sensitivity as it is shown in Alloxan diabetic rats which were administrated by the chloroform extract of the *Musa sapientum* flowers (17). These data show that PE administration ameliorates hyperglycemia and hyperlipidemia in Alloxan-diabetic rats.

## Conclusion

Taken together, the current results show that PE administration ameliorates blood glucose, FFA and TG concentrations levels via insulin sensitivity improvement in Alloxan-induced diabetic Wistar rats. These effects are revealed through the enhancement of insulin expression and secretion of pancreatic β-cells, increased mRNAs expression and activities of IRS-1 and Akt, which result in elevated glucose uptake and promote its storage followed by modulation of the impaired glycolysis and lipolysis in Alloxan-diabetic rats.
